# Was ist gesichert in der Therapie des systemischen Lupus erythematodes?

**DOI:** 10.1007/s00108-023-01624-9

**Published:** 2023-11-07

**Authors:** Vega Gödecke, Torsten Witte

**Affiliations:** 1 Klinik für Nieren- und Hochdruckerkrankungen, Medizinische Hochschule Hannover, Carl-Neuberg-Straße 1, 30625 Hannover, Deutschland; 2 Klinik für Rheumatologie und Immunologie, Medizinische Hochschule Hannover, Carl-Neuberg-Straße 1, 30625 Hannover, Deutschland

**Keywords:** Systemischer Lupus erythematodes/Basismaßnahmen, Biologika, Lupusnephritis, Glukokortikoide, Hydroxychloroquin, Systemic lupus erythematosus/basic treatment measures, Biological therapy, Lupus nephritis, Glucocorticoids, Hydroxychloroquine

## Abstract

Der systemische Lupus erythematodes (SLE) ist eine Autoimmunerkrankung mit variabler klinischer Präsentation und Organbeteiligung. Eine frühzeitige Diagnose sowie das zügige Erreichen einer niedrigen Krankheitsaktivität oder Remission reduzieren Organschäden und verbessern die Prognose. Die Behandlungsansätze lassen sich unterteilen in sogenannte Basismaßnahmen und die immunsuppressiven Therapien. Die medikamentösen Therapieoptionen wurden in den letzten Jahren grundlegend erweitert, dabei sind neue Wirkstoffklassen für die Therapie des SLE hinzugekommen. Dies schließt Biologikatherapien und zugelassene Therapieoptionen für die Behandlung der Lupusnephritis ein. Aufgrund verbesserter Behandlungsmöglichkeiten kann unter Einsparung von Glukokortikoiden häufig eine gute Krankheitskontrolle erreicht werden, dabei kommen zunehmend Kombinationstherapien zum Einsatz. Von großer Wichtigkeit ist der konsequente Einsatz der Basismaßnahmen, hierzu zählen der Einsatz von Hydroxychloroquin, die Optimierung der kardiovaskulären Risikofaktoren, ein Schutz vor ultravioletter Strahlung, knochenprotektive Maßnahmen und die Durchführung der Schutzimpfungen. In der Behandlung der Lupusnephritis spielen konservative Therapiemaßnahmen zur Nephroprotektion eine entscheidende Rolle für die renale Prognose. Hinsichtlich einer Verbesserung der Lebensqualität haben auch nichtpharmakologische Therapieoptionen wie die Bewegungstherapie eine große Bedeutung.

## Abstract (en)

Systemic lupus erythematosus (SLE) is an autoimmune disease with variable clinical presentation and organ involvement. Early diagnosis and rapid achievement of low disease activity or remission reduces organ damage and improves prognosis. Therapeutic principles can be divided into so-called basic measures and immunosuppressive treatment. Novel drugs have been developed in recent years, with new classes of agents being added for the treatment of SLE. These include biologic therapies and approved therapeutic options for the treatment of lupus nephritis. In light of improved treatment options, good disease control can now frequently be achieved; with savings on glucocorticoids, combination therapies are increasingly being used. Of great importance is the consistent use of basic measures, which include the use of hydroxychloroquine, optimization of cardiovascular risk factors, UV protection, bone-protective measures, and the implementation of vaccinations. In the treatment of lupus nephritis, conservative therapeutic measures for nephroprotection play a crucial role in renal prognosis. Finally, non-pharmacological therapy options such as exercise therapy are of great importance for improving quality of life.

Der systemische Lupus erythematodes (SLE) kann mit unterschiedlichen Organmanifestationen einhergehen und wird mit einer Prävalenz von 20 bis 70/100.000 Einwohner zu den seltenen Erkrankungen gezählt [1]. Obwohl sich die diagnostischen und therapeutischen Möglichkeiten in den letzten Jahrzehnten verbessert haben, bleibt die „standardized mortality ratio“ weiterhin erhöht, insbesondere bei juvenilem SLE [2]. Frauen sind deutlich häufiger vom SLE betroffen als Männer, ebenso unterscheidet sich die Prävalenz je nach ethnischer Zugehörigkeit.

## Symptome

Die klinische Präsentation bei Erstdiagnose ist sehr variabel, fast jedes Organ kann betroffen sein. Etwa 40–60 % der Patienten leiden unter einer Nierenbeteiligung, die häufig bereits zu Beginn oder innerhalb der ersten Jahre nach Diagnosestellung auftritt und mit einem erhöhten Risiko für die Entwicklung einer Dialysepflichtigkeit einhergeht [3]. Im Laufe der Erkrankung kann die klinische Symptomatik je nach Krankheitsaktivität variieren. Häufige Symptome sind Fatigue, Myalgien, Arthralgien, Alopezie, orale Ulzera, wie auch Organbeteiligungen der Nieren, Haut, Gefäße oder des Nervensystems.

Art und Schwere der Organbeteiligung haben erheblichen Einfluss auf die Wahl der optimalen Therapie

Bei Verdacht auf einen SLE sollten eine komplette Anamnese und klinische Untersuchung durchgeführt werden, um mögliche Organmanifestationen zu erkennen. Art und Schwere der Organbeteiligung haben erheblichen Einfluss auf die Auswahl der optimalen Therapie. Eine frühe Diagnose, insbesondere einer Nierenbeteiligung, ist prognostisch relevant. Die Diagnose wird auf Grundlage von klinischen, serologischen, gegebenenfalls bildgebenden und/oder histologischen Befunden gestellt. Die SLE Klassifikationskriterien wurden 2019 überarbeitet [4].

## Krankheitsaktivität

Die Beurteilung der Krankheitsaktivität spielt

- bei der Einschätzung des Verlaufs,
- bei der Entscheidung über weitere diagnostische Maßnahmen sowie
- vor einer Modifikation der Therapie

eine zentrale Rolle. Die Krankheitsaktivität sollte daher bei jeder Verlaufskontrolle mit einem standardisierten Aktivitätsscore erhoben werden, beispielsweise mit dem SLE Disease Activity Index 2000 (SLEDAI-2K) [5], dem Physician’s Global Assessment (PGA) oder dem British-Isles-Lupus-Assessment-Group 2004 (BILAG-2004)-Index [6]. Ein Zustand niedriger SLE-Aktivität („lupus low disease activity state“ [LLDAS]) ist definiert als SLEDAI-2K ≤ 4, PGA ≤ 1 und keine neue Lupusaktivität bzw. das Fehlen von relevanter Organaktivität unter maximal 7,5 mg Prednisolon täglich sowie unter einer möglichen immunsuppressiven Erhaltungstherapie [7]. Eine komplette Remission ist erreicht bei SLEDAI-2K = 0 und PGA ≤ 0,5 unter maximal 5 mg Prednisolon täglich sowie unter einer möglichen immunsuppressiven Erhaltungstherapie und Hydroxychloroquin [8].

## Therapie

**Figure 1:**
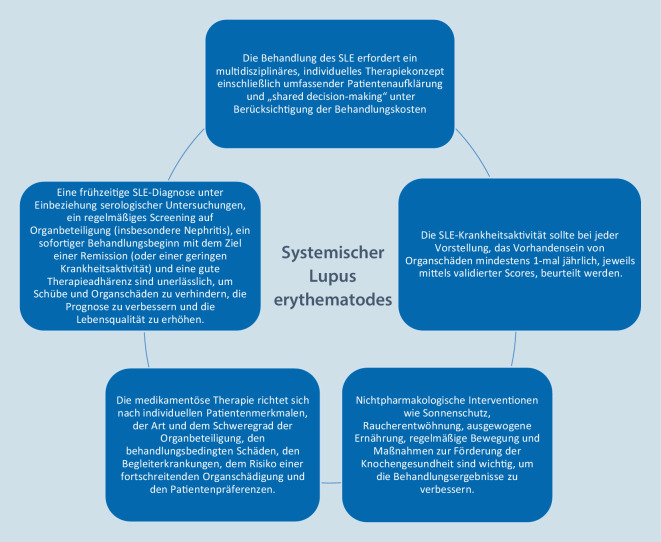

Nachdem die Diagnose SLE gestellt wurde, sollte eine an die Organmanifestationen, Komorbiditäten und individuellen Patientenfaktoren angepasste Therapie eingeleitet werden (Abb. 1). Es sollten die Antiphospholipidantikörper bestimmt und das kardiovaskuläre und infektiologische Risikoprofil erhoben werden, um die Therapie entsprechend anzupassen. Ziel der Therapie ist eine komplette Remission oder ein Zustand niedriger SLE-Aktivität (LLDAS). Der Einsatz von Glukokortikoiden sollte wenn möglich zeitlich begrenzt werden, um unerwünschte Arzneimittelwirkungen zu minimieren.

Bei der Therapie muss zwischen sogenannten Basismaßnahmen und einer immunmodulierenden bzw. immunsuppressiven Therapie unterschieden werden. Zu den Basismaßnahmen gehören die Therapie mit Antimalariamitteln, der Schutz vor ultravioletter (UV) Strahlung, die Durchführung der indizierten Schutzimpfungen, der Knochenschutz und die Erhebung und Modifikation der kardiovaskulären Risikofaktoren. Als Erinnerungshilfe bietet sich das englische Akronym BASIC für

- B – „bones“ (Knochen),
- A – „antimalarial“,
- S – „sun protection“ (Sonnenschutz),
- I – „immunization“ (Impfung) und
- C – „cardiovascular“

an [10]. Weiterhin sehen die Empfehlungen der European Alliance of Associations for Rheumatology (EULAR) von 2023 vor, dass nichtpharmakologische Therapiestrategien zum festen Bestandteil der SLE-Therapie werden [11]. In randomisierten Studien konnte gezeigt werden, dass sowohl physisches Training als auch psychologische Unterstützung die Lebensqualität bei SLE verbessern [12, 13]. Absolute Nikotinkarenz wird dringend empfohlen, unter anderem da Nikotinkonsum das Ansprechen sowohl auf Hydroxychloroquin als auch auf Belimumab vermindert [14].

Physisches Training wie auch psychologische Unterstützung verbessern die Lebensqualität bei SLE

Die immunsuppressive Therapie orientiert sich an der Schwere der Krankheitsmanifestation und am klinischen Verlauf, aber auch an Patientenfaktoren, Präferenzen und Komorbidität. Laut den aktuellen Empfehlungen der europäischen Fachgesellschaft EULAR von 2023 [9] wird bei den Therapieempfehlungen zwischen extrarenalem SLE und renalem SLE unterschieden. Bei extrarenalem SLE mit leichtem Verlauf, etwa mit Allgemeinsymptomen, Haut- oder Gelenkbeteiligung, wird eine Therapie mit dem Antimalariamittel Hydroxychloroquin und niedrig dosierten Glukokortikoiden empfohlen. Bei fehlendem Ansprechen bzw. bei moderaten Verläufen mit ausgeprägterer Haut- oder Gelenkbeteiligung, Thrombozytopenie oder Serositis wird zusätzlich Mycophenolatmofetil (MMF), Azathioprin oder Methotrexat, gegebenenfalls in Kombination mit Belimumab oder Anifrolumab, empfohlen. Bei moderaten Verläufen kommen auch Calcineurininhibitoren in Betracht. Bei schweren Verläufen mit Beteiligung innerer Organe oder des zentralen Nervensystems (ZNS) ist eine Therapie mit initial hoch dosierten Glukokortikoiden, Cyclophosphamid, MMF bzw. in refraktären Fällen Rituximab indiziert. Anifrolumab oder Belimumab wird zusätzlich bei schwerem extrarenalem SLE mit ausgedehnter Erkrankung von Haut oder Gelenken eingesetzt. Bei renalem SLE können zusätzlich zu Hydroxychloroquin und Glukokortikoiden folgende Therapieschemata eingesetzt werden: Zum einen initial Cyclophosphamid gefolgt von Azathioprin oder MMF, zum anderen MMF in der Initial- und Folgetherapie. Dabei kann Belimumab als zusätzliche Therapie bei allen Therapieschemata eingesetzt werden, für Voclosporin besteht eine Zulassung in der Kombinationstherapie mit MMF. Bei rezidivierender bzw. refraktärer Erkrankung, insbesondere nach Versagen von cyclophosphamidbasierten Therapien, kommt auch eine Therapie mit Rituximab in Betracht.

### Wirkstoffe

#### Glukokortikoide

Der Einsatz von Glukokortikoiden ist als Initialtherapie und bei Zunahme der Krankheitsaktivität etabliert. Die initiale Dosierung ist dabei abhängig von den Organmanifestationen. Die Dosis sollte langfristig so niedrig wie möglich gehalten werden, es wird eine Prednisolondosis von maximal 5 mg täglich in der Erhaltungstherapie angestrebt, sofern ein Ausschleichen nicht möglich ist.

#### Hydroxychloroquin

Das Antimalariamittel Hydroxychloroquin hat sich als Standardtherapeutikum etabliert und sollte, sofern keine relevanten Kontraindikationen vorliegen, bei allen Patienten mit SLE zum Einsatz kommen, oft in Kombination mit anderen Medikamenten [9]. Unter Hydroxychloroquin bestehen eine verminderte Schubrate und eine bessere Langzeitprognose [15, 16]. Unter Einhaltung der empfohlenen Höchstdosis von 5 mg/kg pro Tag ist das Risiko der Entwicklung einer Retinopathie gering, es werden jedoch unter der Therapie regelmäßige ophthalmologische Kontrolluntersuchungen empfohlen.

#### Mycophenolatmofetil

MMF gehört zu den Standardtherapeutika bei der Lupusnephritis. Es ist zwar formal nicht für die SLE-Therapie zugelassen, wurde jedoch vom Gemeinsamen Bundesausschuss für die Therapie einer Lupusnephritis der Klasse III–V nach Weltgesundheitsorganisation (WHO) empfohlen, bei Klasse III und IV als Alternative zu Cyclophosphamid. In der Induktionstherapie ist es einer Cyclophosphamidbolustherapie gleichwertig und in der Erhaltungstherapie Azathioprin überlegen. Bei starker Krankheitsaktivität oder refraktärem Verlauf bietet sich eine Kombination mit Biologikatherapien oder Voclosporin plus jeweils Hydroxychloroquin an. Die meisten Studien wurden bei Patienten mit Lupusnephritis durchgeführt, es zeigte sich jedoch unter MMF auch eine Verbesserung der globalen Krankheitsaktivität, insbesondere ein positiver Effekt auf mukokutane und muskuloskeletale Manifestationen [17]. Dabei zeigte sich bei SLE ohne renale Beteiligung unter MMF-Therapie eine höhere Remissionsrate als unter Azathioprin [18], daher kommt MMF auch bei therapierefraktärem nichtrenalem SLE in Betracht [19].

#### Belimumab

Belimumab ist ein humaner monoklonaler Antikörper gegen das lösliche B‑Zell-Zytokin B‑Lymphozyten-Stimulator (BLyS) bzw. B‑Lymphozyten-aktivierender Faktor (BAFF) und zeigte beim extrarenalen SLE im Rahmen der Zulassungsstudien einen guten Effekt hinsichtlich einer Reduktion von Krankheitsschüben, Aktivität, Organschäden und Steroidgebrauch, insbesondere bei frühem Einsatz [20, 21]. Die Indikation besteht bei klinischer sowie serologischer Aktivität des SLE (Antikörper gegen doppelsträngige DNA [dsDNA-Ak], Komplementverbrauch). Auch beim renalen SLE ist Belimumab inzwischen zugelassen; es zeigte eine Verbesserung der renalen Endpunkte Proteinurie und Nierenfunktion [22], Eine Kombinationstherapie mit Belimumab kann beim renalen Lupus bereits ab Diagnosestellung erfolgen.

#### Anifrolumab

Anifrolumab ist ein Typ-I-Interferon-Rezeptor-Antikörper und wird beim moderaten bis schweren aktiven SLE zusätzlich zur Standardtherapie eingesetzt. Anifrolumab zeigte in einer der Zulassungsstudien einen guten Effekt insbesondere auf Hautmanifestationen, so dass Glukokortikoide reduziert werden konnten [23]. Dieser Effekt hat sich in den Langzeitdaten bestätigt [24]. Eine Zulassung für die Therapie der Lupusnephritis besteht bislang nicht. Indikationsimpfungen vor Beginn der Therapie werden empfohlen.

#### Voclosporin

Voclosporin ist ein neuartiger Calcineurininhibitor, der in Kombination mit MMF zur Behandlung der Lupusnephritis zugelassen ist. In der Zulassungsstudie und in den Verlaufsdaten [25, 26] zeigte sich ein guter Effekt auf die Reduktion der Proteinurie. Dabei spielen sowohl die immunsuppressiven als auch die podozytenstabilisierenden Effekte einer Calcineurininhibitortherapie eine Rolle. Im Vergleich zu anderen Calcineurininhibitoren sind Serumspiegelbestimmungen nicht erforderlich, es sollte jedoch initial und im Verlauf eine Kontrolle der Nierenfunktion und je nach Ergebnissen gegebenenfalls eine Dosisanpassung erfolgen. In der Zulassungsstudie war eine geschätzte glomeruläre Filtrationsrate von >45 ml/min/1,73 m^2^ Körperoberfläche nach CKD-EPI (Chronic Kidney Disease Epidemiology Collaboration) Voraussetzung für den Studieneinschluss [20], für den Einsatz bei schwerer Nierenfunktionseinschränkung liegen bislang keine Daten vor.

#### Cyclophosphamid

Cyclophosphamid ist als Zytostatikum zur Behandlung der Lupusnephritis zugelassen und wird aufgrund des besseren Nutzen-Nebenwirkungs-Verhältnisses aktuell zumeist in der Dosis nach Euro-Lupus-Schema [27] eingesetzt. Weitere Indikationen für eine Cyclophosphamidtherapie sind schwere Organbeteiligungen wie ZNS- oder Lungenbeteiligung.

#### Azathioprin

Azathioprin ist für die Therapie des SLE zugelassen und kommt bei leichten bis moderaten Krankheitsverläufen zum Einsatz, alternativ bei Lupusnephritis in der Erhaltungstherapie nach Induktion mit Cyclophosphamid. Azathioprin ist bei SLE ohne renale Beteiligung MMF im direkten Vergleich unterlegen [18]. Daher erfolgt die Gabe von Azathioprin bevorzugt in bestimmten Situationen wie etwa bei Schwangerschaftswunsch.

#### Methotrexat

Methotrexat ist nicht für die Indikation SLE zugelassen, kommt aber insbesondere bei leichten bis mittelschweren Krankheitsverläufen mit muskuloskeletaler und dermatologischer Beteiligung zum Einsatz und hat hier einen guten Effekt auf die Gelenkbeteiligung und die globale Krankheitsaktivität [28]. Zu beachten ist, dass eine fortgeschrittene Niereninsuffizienz eine Kontraindikation darstellt.

#### Rituximab

Wenngleich randomisierte Studien für Rituximab bei Lupusnephritis keinen signifikanten Effekt zusätzlich zu einer MMF-Therapie belegen konnten [29], zeigte sich doch eine Verbesserung der immunserologischen Parameter. Eine Metaanalyse ergab, dass unter Rituximab bei SLE die Krankheitsaktivität deutlich abnahm und die Glukokortikoiddosis reduziert werden konnte, bei Lupusnephritis kam es zu einer signifikanten Reduktion der Proteinurie [30]. In den aktuellen EULAR-Leitlinien von 2023 wird daher der Off-label-Einsatz von Rituximab bei schwerem und auch therapierefraktärem Verlauf oder ausgeprägter Immunthrombozytopenie aufgeführt, bei Lupusnephritis insbesondere nach Versagen einer cyclophosphamidbasierten Therapie [9].

### Therapie bei systemischem Lupus erythematodes und Schwangerschaft

Vor Eintritt einer Schwangerschaft wird eine Remission der Grunderkrankung angestrebt, um das Risiko für Schwangerschaftskomplikationen und einen SLE-Schub zu senken. Ein erniedrigtes Serum-C4 vor Eintritt einer Schwangerschaft zeigt ein erhöhtes Risiko für einen Schub der Grunderkrankung im Schwangerschaftsverlauf an [31]. Die Überwachung der serologischen Parameter (Komplementfaktoren, dsDNA-Ak) sowie auch nephrologischer Marker (S-Kreatinin, Proteinurie, Urinsediment) sowie eine engmaschige gynäkologische Überwachung nach Eintritt der Schwangerschaft werden empfohlen.

Die Fortsetzung einer Hydroxychloroquintherapie in der Schwangerschaft erbrachte positive Effekte und senkte das Risiko für Schübe [32], zusätzlich kommt bei erhöhtem Präeklampsierisiko niedrig dosierte Acetylsalicylsäure und je nach Risikoprofil niedermolekulares Heparin zum Einsatz. Als immunsuppressive Therapeutika kommen in der Schwangerschaft Azathioprin, konventionelle Calcineurininhibitoren und/oder Glukokortikoide infrage. Bei geplanter Schwangerschaft sollte eine Evaluation und ggf. Umstellung der medikamentösen Therapie bereits im Vorfeld erfolgen. Daher wird empfohlen, das Thema Schwangerschaftswunsch bei Frauen im gebärfähigen Alter so früh wie möglich nach Diagnosestellung und auch im Verlauf regelmäßig anzusprechen [32].

Bei geplanter Schwangerschaft sollte die medikamentöse Therapie bereits im Vorfeld evaluiert und ggf. umgestellt werden

### Therapie des systemischen Lupus erythematodes im Wandel

Die Entwicklung und Zulassung neuer Therapien, insbesondere Biologikatherapien, stellt einen Fortschritt in der Behandlung des SLE dar und erlaubt den Beginn einer Kombinationstherapie bereits ab Diagnosestellung. Idealerweise ist hierunter ein zügiges Ausschleichen der Glukokortikoide bei effektiver Reduktion der Krankheitsaktivität und Erreichen einer Remission möglich. Prednisolon sollte idealerweise nur im Rahmen der Erstmanifestation und bei Schüben zum Einsatz kommen und langfristig maximal in einer Dosierung von 5 mg täglich gegeben werden, ein Absetzen wird angestrebt. Bei moderater bis schwerer Krankheitsaktivität kann eine Pulstherapie mit Methylprednisolon in Erwägung gezogen werden. Hydroxychloroquin ist das Therapeutikum der Wahl beim SLE, sofern keine Kontraindikationen bestehen. Die Krankheitsaktivität sollte regelmäßig mittels validierter Scores erfasst und die Therapie entsprechend angepasst werden. Auf nichtpharmakologische Therapieansätze sowie die Optimierung kardiovaskulärer Risikofaktoren sollte geachtet werden.

Bei Nierenbeteiligung sind sowohl Belimumab in Kombination mit einer immunsuppressiven Basistherapie als auch Voclosporin in Kombination mit MMF zugelassen. Zudem kommen bei Nierenbeteiligung nephroprotektive Substanzen wie Angiotensin-converting-enzyme(ACE)-Hemmer oder Angiotensin-II-Rezeptor-Blocker zum Einsatz, für Natrium-Glukose-Kotransporter-2(SGLT-2)-Hemmer besteht aktuell eine Zulassung bei entsprechender Nierenfunktionseinschränkung.

Dem erhöhten kardiovaskulären Risiko und Osteoporoserisiko sollte Rechnung getragen werden, zudem werden neben dem Schutz vor UV-Strahlung regelmäßige Krebsvorsorgeuntersuchungen und Schutzimpfungen empfohlen. Bei Schwangerschaftswunsch wird eine frühzeitige Evaluation in einer Spezialsprechstunde empfohlen, um die SLE-Therapie planen und die Risiken minimieren zu können.

Zu bedenken ist, dass trotz verbesserter Therapieoptionen eine Remission nicht in allen Fällen zu erreichen ist und insbesondere in diesen Fällen ein individuelles Therapiekonzept notwendig ist. Es sollte im Rahmen der Verlaufskontrollen über die zuverlässige Einnahme der Medikation gesprochen und bei fehlender Adhärenz die Behandlungsstrategie angepasst werden.

Die aktuellen EULAR-Leitlinien von 2023 fassen diese Empfehlungen zur Therapie des SLE zusammen [9]. Sie sind in übergeordnete Therapieprinzipien (Abb. 1) und konkrete Behandlungsempfehlungen (Tab. 1) unterteilt.

**Table 1:** 

Medikamentöse Therapie	Hydroxychloroquin wird für alle Patienten (1b/A) empfohlen, außer bei Kontraindikationen. Die Zieldosis beträgt 5 mg/kg reales Körpergewicht pro Tag (2b/B) und kann je nach Risiko eines Krankheitsschubs (2b/B) und Risiko einer retinalen Toxizität individuell angepasst werden
	Bei Patienten, die nicht auf Hydroxychloroquin (allein oder in Kombination mit Glukokortikoiden) ansprechen, oder bei Patienten, bei denen die Glukokortikoide nicht auf eine akzeptable Dosis reduziert werden können, sollte die Gabe von immunmodulierenden/immunsuppressiven Mitteln, z. B. Methotrexat (1b/B), Azathioprin (2b/C) oder Mycophenolat (2a/B), und/oder Biologika, z. B. Belimumab (1a/A) oder Anifrolumab (1a/A), erwogen werden
	Bei Patienten mit organbedrohenden oder lebensbedrohlichen Erkrankungen kommt der Einsatz von intravenösem Cyclophosphamid (2b/C) infrage; in refraktären Fällen kann Rituximab (2b/C) in Betracht gezogen werden
	Glukokortikoide werden, falls erforderlich, je nach Art und Schwere der Organbeteiligung dosiert (2b/C) und sollten auf eine Erhaltungsdosis von ≤ 5 mg/Tag (Prednisonäquivalent; 2a/B) reduziert und, wenn möglich, abgesetzt werden; bei Patienten mit mittelschwerer bis schwerer Erkrankung können Pulse von intravenösem Methylprednisolon (125–1000 mg pro Tag, für 1–3 Tage; 3b/C) in Betracht gezogen werden
	Bei Patienten mit SLE, die eine anhaltende Remission erreicht haben, sollte ein schrittweises Absetzen der Behandlung erwogen werden, wobei zunächst die Glukokortikoide abgesetzt werden sollten (2a/B)
Lupusnephritis	Patienten mit aktiver, proliferativer Lupusnephritis sollten niedrig dosiertes (Euro-Lupus-Schema) intravenöses Cyclophosphamid (1a/A) oder Mycophenolat (1a/A) und Glukokortikoide (Pulse von intravenösem Methylprednisolon gefolgt von niedrigeren oralen Dosen) erhalten; eine Kombinationstherapie mit Belimumab (entweder mit Cyclophosphamid oder mit Mycophenolat; 1b/A) oder Calcineurininhibitoren (insbesondere Voclosporin oder Tacrolimus, kombiniert mit Mycophenolat; 1b/A) sollte erwogen werden
	Nach dem renalen Ansprechen sollte die Behandlung der Lupusnephritis für mindestens 3 Jahre fortgesetzt werden (2b/B); Patienten, die ursprünglich mit Mycophenolat allein oder in Kombination mit Belimumab oder einem Calcineurininhibitor behandelt wurden, sollten diese Medikamente weiter einnehmen (1a/A), während Mycophenolat oder Azathioprin das Cyclophosphamid bei den Patienten ersetzen sollte, die ursprünglich mit Cyclophosphamid allein (1a/A) oder in Kombination mit Belimumab (1a/A) behandelt wurden
	Bei Patienten mit einem hohen Risiko für ein Nierenversagen (definiert als reduzierte glomeruläre Filtrationsrate, histologisches Vorhandensein von zellulären Halbmonden oder fibrinoiden Nekrosen oder schwere interstitielle Entzündungen) kann hoch dosiertes (National-Institutes-of-Health-Regime) intravenöses Cyclophosphamid (1a/A) in Kombination mit gepulstem intravenösem Methylprednisolon in Betracht gezogen werden
Hautbeteiligung	Die Behandlung einer aktiven Hauterkrankung sollte je nach Bedarf topische Mittel (Glukokortikoide, Calcineurininhibitoren; 2b/B), Antimalariamittel (Hydroxychloroquin, Chloroquin; 1a/A) und/oder systemische Glukokortikoide (4/C) umfassen, wobei Anifrolumab (1a/A), Belimumab (1a/B), Methotrexat (1b/B) oder Mycophenolat (4/C) als Zweitlinientherapie infrage kommen
Immunthrombozytopenie	Zur akuten Behandlung einer schweren Autoimmunthrombozytopenie sollten hoch dosierte Glukokortikoide (einschließlich intravenöser Methylprednisolonpulse; 4/C), mit oder ohne intravenöses Immunglobulin G (4/C), und/oder Rituximab (2b/B) und/oder hoch dosiertes intravenöses Cyclophosphamid (4/C) in Betracht gezogen werden, gefolgt von einer Erhaltungstherapie mit Rituximab (2b/B), Azathioprin (2b/C), Mycophenolat (2b/C) oder Cyclosporin (4/C)
Neuropsychiatrische Beteiligung	Bei aktiven neuropsychiatrischen Erkrankungen, die auf einen SLE zurückzuführen sind, sollten Glukokortikoide und Immunsuppressiva bei entzündlichen Manifestationen (1b/A) und Thrombozytenaggregationshemmer/Antikoagulanzien bei atherothrombotischen/aPL-bedingten Manifestationen (2b/C) in Betracht gezogen werden
Antiphospholipidsyndrom	Ein APS im Rahmen eines SLE sollte nach dem ersten arteriellen oder spontan aufgetretenen venösen thrombembolischen Ereignis mit Vitamin-K-Antagonisten behandelt werden (1b/B); niedrig dosierte Acetylsalicylsäure (75–100 mg/Tag) sollte bei Patienten mit SLE ohne APS, aber mit hohem aPL-Risikoprofil in Betracht gezogen werden (2a/B)
Sonstiges	Schutzimpfungen (u. a. Herpes-zoster-Virus, humanes Papillomavirus, Influenza, COVID-19 und Pneumokokken), osteoprotektive und nephroprotektive Maßnahmen, eine Reduktion kardiovaskulärer Risikofaktoren sowie die Durchführung von Krebsvorsorgeuntersuchungen werden empfohlen (5/D)

Evidenzlevel siehe „Oxford evidence-based medicine grading levels“ (https://www.cebm.net/wp-content/uploads/2014/06/CEBM-Levels-of-Evidence-2.1.pdf)

*aPL* Antiphospholipidantikörper, *APS* Antiphospholipidsyndrom, *COVID-19* „coronavirus disease 2019“, *SLE* systemischer Lupus erythematodes

## Fazit für die Praxis

- Der systemische Lupus erythematodes (SLE) ist eine chronische Multisystemerkrankung, die aufgrund klinischer Befunde und charakteristischer serologischer Parameter diagnostiziert wird.
- Neben Basismaßnahmen wie dem Schutz vor ultravioletter Strahlung, Knochenschutz, Impfungen, Reduktion der kardiovaskulären Risikofaktoren sowie Nikotinkarenz besteht bei SLE eine Indikation für Hydroxychloroquin, zudem sollten nichtpharmakologische Interventionen erfolgen.
- Je nach Organbeteiligung kommen verschiedene immunsuppressive Strategien zum Einsatz. Als Kombinationspartner stehen für den SLE zugelassene Biologika wie Belimumab oder Anifrolumab zur Verfügung, für die Lupusnephritis sind Voclosporin und Belimumab zugelassen.
- Der schwere SLE erfordert zumeist eine interdisziplinäre Behandlung. Nach anfänglich intensiver immunsuppressiver Therapie zur Kontrolle der Krankheitsaktivität folgt eine immunsuppressive Erhaltungstherapie.
- Die Behandlung sollte auf eine Remission oder geringe Krankheitsaktivität und die Verhinderung von Krankheitsschüben in allen Organen abzielen, unter Einsparung von Glukokortikoiden (Ziel: Prednisolon ≤ 5 mg täglich).
- Zu den Behandlungszielen gehören eine Prognoseverbesserung, die Vermeidung von Organschäden und die Optimierung der gesundheitsbezogenen Lebensqualität.

## References

[1] Pons-Estel GJ, Alarcón GS, Scofield L, Reinlib L, Cooper GS (2010). Understanding the epidemiology and progression of systemic lupus erythematosus. Semin Arthritis Rheum.

[2] Moe SR, Haukeland H, Brunborg C, Botea A, Damjanic N, Wivestad GÅ, Øvreås H, Bøe T, Orre A, Garen T, Lilleby V, Provan SA, Molberg Ø, Lerang K (2023). Persisting mortality gap in systemic lupus erythematosus; a population-based study on juvenile- and adult-onset SLE in Norway 1999–2022. Rheumatology.

[3] Anders HJ, Saxena R, Zhao MH, Parodis I, Salmon JE, Mohan C (2020). Lupus nephritis. Nat Rev Dis Primers.

[4] Aringer M, Costenbader K, Daikh D, Brinks R, Mosca M, Ramsey-Goldman R, Smolen JS, Wofsy D, Boumpas DT, Kamen DL, Jayne D, Cervera R, Costedoat-Chalumeau N, Diamond B, Gladman DD, Hahn B, Hiepe F, Jacobsen S, Khanna D, Lerstrøm K, Massarotti E, McCune J, Ruiz-Irastorza G, Sanchez-Guerrero J, Schneider M, Urowitz M, Bertsias G, Hoyer BF, Leuchten N, Tani C, Tedeschi SK, Touma Z, Schmajuk G, Anic B, Assan F, Chan TM, Clarke AE, Crow MK, Czirják L, Doria A, Graninger W, Halda-Kiss B, Hasni S, Izmirly PM, Jung M, Kumánovics G, Mariette X, Padjen I, Pego-Reigosa JM, Romero-Diaz J, Rúa-Figueroa Fernández Í, Seror R, Stummvoll GH, Tanaka Y, Tektonidou MG, Vasconcelos C, Vital EM, Wallace DJ, Yavuz S, Meroni PL, Fritzler MJ, Naden R, Dörner T, Johnson SR (2019). 2019 European League Against Rheumatism/American College of Rheumatology classification criteria for systemic lupus erythematosus. Ann Rheum Dis.

[5] Gladman DD, Ibañez D, Urowitz MB (2000). Systemic lupus erythematosus disease activity index 2000. J Rheumatol.

[6] Yee CS, Farewell V, Isenberg DA, Rahman A, Teh LS, Griffiths B, Bruce IN, Ahmad Y, Prabu A, Akil M, McHugh N, D’Cruz D, Khamashta MA, Maddison P, Gordon C (2004). British Isles Lupus Assessment Group 2004 index is valid for assessment of disease activity in systemic lupus erythematosus. Arthritis Rheum..

[7] Franklyn K, Lau CS, Navarra SV, Louthrenoo W, Lateef A, Hamijoyo L, Wahono CS, Chen SL, Jin O, Morton S, Hoi A, Huq M, Nikpour M, Morand EF (2016). Asia-pacific lupus collaboration. Definition and initial validation of a lupus low disease activity state (LLDAS). Ann Rheum Dis.

[8] van Vollenhoven RF, Bertsias G, Doria A, Isenberg D, Morand E, Petri MA, Pons-Estel BA, Rahman A, Ugarte-Gil MF, Voskuyl A, Arnaud L, Bruce IN, Cervera R, Costedoat-Chalumeau N, Gordon C, Houssiau FA, Mosca M, Schneider M, Ward MM, Alarcon G, Aringer M, Askenase A, Bae SC, Bootsma H, Boumpas DT, Brunner H, Clarke AE, Coney C, Czirják L, Dörner T, Faria R, Fischer R, Fritsch-Stork R, Inanc M, Jacobsen S, Jayne D, Kuhn A, van Leeuw B, Limper M, Mariette X, Navarra S, Nikpour M, Olesinska MH, Pons-Estel G, Romero-Diaz J, Rubio B, Schoenfeld Y, Bonfá E, Smolen J, Teng YKO, Tincani A, Tsang-A-Sjoe M, Vasconcelos C, Voss A, Werth VP, Zakharhova E, Aranow C (2021). 2021 DORIS definition of remission in SLE: final recommendations from an international task force. Lupus Sci Med.

[9] Fanouriakis A, Kostopoulou M, Andersen J, Aringer M, Arnaud L, Bae SC, Boletis J, Bruce IN, Cervera R, Doria A, Dörner T, Furie RA, Gladman DD, Houssiau FA, Inês LS, Jayne D, Kouloumas M, Kovács L, Mok CC, Morand EF, Moroni G, Mosca M, Mucke J, Mukhtyar CB, Nagy G, Navarra S, Parodis I, Pego-Reigosa JM, Petri M, Pons-Estel BA, Schneider M, Smolen JS, Svenungsson E, Tanaka Y, Tektonidou MG, Teng YO, Tincani A, Vital EM, van Vollenhoven RF, Wincup C, Bertsias G, Boumpas DT (2023). EULAR recommendations for the management of systemic lupus erythematosus: 2023 update. Ann Rheum Dis.

[10] Aringer M, Schneider M (2016). Management des systemischen Lupus erythematodes [Management of systemic lupus erythematosus]. Internist.

[11] Parodis I, Girard-Guyonvarc’h C, Arnaud L, Distler O, Domján A, Van den Ende CHM, Fligelstone K, Kocher A, Larosa M, Lau M, Mitropoulos A, Ndosi M, Poole JL, Redmond A, Ritschl V, Alexanderson H, Sjöberg Y, von Perner G, Uhlig T, Varju C, Vriezekolk JE, Welin E, Westhovens R, Stamm TA, Boström C (2023). EULAR recommendations for the non-pharmacological management of systemic lupus erythematosus and systemic sclerosis. Ann Rheum Dis.

[12] Liang H, Tian X, Cao LY, Chen YY, Wang CM (2014). Effect of psychological intervention on health-related quality of life in people with systemic lupus erythematosus: a systematic review. Int J Nurs Sci.

[13] da Hora TC, Lima K, Maciel RRBT (2019). The effect of therapies on the quality of life of patients with systemic lupus erythematosus: a meta-analysis of randomized trials. Adv Rheumatol.

[14] Parisis D, Bernier C, Chasset F, Arnaud L (2019). Impact of tobacco smoking upon disease risk, activity and therapeutic response in systemic lupus erythematosus: A systematic review and meta-analysis. Autoimmun Rev.

[15] Canadian Hydroxychloroquine Study Group (1991). A randomized study of the effect of withdrawing hydroxychloroquine sulfate in systemic lupus erythematosus. N Engl J Med.

[16] Ruiz-Irastorza G, Ramos-Casals M, Brito-Zeron P, Khamashta MA (2010). Clinical efficacy and side effects of antimalarials in systemic lupus erythematosus: a systematic review. Ann Rheum Dis.

[17] Ginzler EM, Wofsy D, Isenberg D, Gordon C, Lisk L, Dooley MA, ALMS Group (2010). Nonrenal disease activity following mycophenolate mofetil or intravenous cyclophosphamide as induction treatment for lupus nephritis: findings in a multicenter, prospective, randomized, open-label, parallel-group clinical trial. Arthritis Rheum.

[18] Ordi-Ros J, Sáez-Comet L, Pérez-Conesa M, Vidal X, Mitjavila F, Castro SA, Cuquet Pedragosa J, Ortiz-Santamaria V, Plana MM, Cortés-Hernández J (2017). Enteric-coated mycophenolate sodium versus azathioprine in patients with active systemic lupus erythematosus: a randomised clinical trial. Ann Rheum Dis.

[19] Aringer M, Fischer-Betz R, Hiepe F, Kommission Pharmakotherapie der DGRh Germany Society of Rheumatology (2013). Stellungnahme zum Einsatz von Mycophenolat-Mofetil beim systemischen Lupus erythematodes [Statement on the use of mycophenolate mofetil for systemic lupus erythematosus]. Z Rheumatol.

[20] Navarra SV, Guzmán RM, Gallacher AE, Hall S, Levy RA, Jimenez RE, Li EK, Thomas M, Kim HY, León MG, Tanasescu C, Nasonov E, Lan JL, Pineda L, Zhong ZJ, Freimuth W, Petri MA, BLISS-52 Study Group (2011). Efficacy and safety of belimumab in patients with active systemic lupus erythematosus: a randomised, placebo-controlled, phase 3 trial. Lancet.

[21] Furie R, Petri M, Zamani O, Cervera R, Wallace DJ, Tegzová D, Sanchez-Guerrero J, Schwarting A, Merrill JT, Chatham WW, Stohl W, Ginzler EM, Hough DR, Zhong ZJ, Freimuth W, van Vollenhoven RF, BLISS-76 Study Group (2011). A phase III, randomized, placebo-controlled study of belimumab, a monoclonal antibody that inhibits B lymphocyte stimulator, in patients with systemic lupus erythematosus. Arthritis Rheum.

[22] Furie R, Rovin BH, Houssiau F, Malvar A, Teng YKO, Contreras G, Amoura Z, Yu X, Mok CC, Santiago MB, Saxena A, Green Y, Ji B, Kleoudis C, Burriss SW, Barnett C, Roth DA (2020). Two-year, randomized, controlled trial of belimumab in lupus nephritis. N Engl J Med.

[23] Morand EF, Furie R, Tanaka Y, Bruce IN, Askanase AD, Richez C, Bae SC, Brohawn PZ, Pineda L, Berglind A, Tummala R, TULIP-2 Trial Investigators (2020). Trial of Anifrolumab in Active Systemic Lupus Erythematosus. N Engl J Med.

[24] Kalunian KC, Furie R, Morand EF, Bruce IN, Manzi S, Tanaka Y, Winthrop K, Hupka I, Zhang LJ, Werther S, Abreu G, Hultquist M, Tummala R, Lindholm C, Al-Mossawi H (2023). A randomized, placebo-controlled phase III extension trial of the long-term safety and tolerability of anifrolumab in active systemic lupus erythematosus. Arthritis Rheumatol.

[25] Rovin BH, Teng YKO, Ginzler EM, Arriens C, Caster DJ, Romero-Diaz J, Gibson K, Kaplan J, Lisk L, Navarra S, Parikh SV, Randhawa S, Solomons N, Huizinga RB (2021). Efficacy and safety of voclosporin versus placebo for lupus nephritis (AURORA 1): a double-blind, randomised, multicentre, placebo-controlled, phase 3 trial. Lancet.

[26] Saxena A, Ginzler EM, Gibson K, Satirapoj B, Santillán AEZ, Levchenko O, Navarra S, Atsumi T, Yasuda S, Chavez-Perez NN, Arriens C, Parikh SV, Caster DJ, Birardi V, Randhawa S, Lisk L, Huizinga RB, Teng YKO (2023). Safety and efficacy of long-term Voclosporin treatment for lupus nephritis in the phase 3 AURORA 2 clinical trial. Arthritis Rheumatol.

[27] Houssiau FA, Vasconcelos C, D’Cruz D, Sebastiani GD, de Ramon Garrido E, Danieli MG, Abramovicz D, Blockmans D, Mathieu A, Direskeneli H, Galeazzi M, Gül A, Levy Y, Petera P, Popovic R, Petrovic R, Sinico RA, Cattaneo R, Font J, Depresseux G, Cosyns JP, Cervera R (2004). Early response to immunosuppressive therapy predicts good renal outcome in lupus nephritis: lessons from long-term followup of patients in the Euro-Lupus Nephritis Trial. Arthritis Rheum.

[28] Carneiro JR, Sato EI (1999). Double blind, randomized, placebo controlled clinical trial of methotrexate in systemic lupus erythematosus. J Rheumatol.

[29] Rovin BH, Furie R, Latinis K, Looney RJ, Fervenza FC, Sanchez-Guerrero J, Maciuca R, Zhang D, Garg JP, Brunetta P, Appel G, LUNAR Investigator Group (2012). Efficacy and safety of rituximab in patients with active proliferative lupus nephritis: the Lupus Nephritis Assessment with Rituximab study. Arthritis Rheum.

[30] Alshaiki F, Obaid E, Almuallim A, Taha R, El-Haddad H, Almoallim H (2018). Outcomes of rituximab therapy in refractory lupus: a meta-analysis. Eur J Rheumatol.

[31] Crisafulli F, Andreoli L, Zucchi D, Reggia R, Gerardi MC, Lini D, Tani C, Zatti S, Franceschini F, Mosca M, Tincani A (2023). Variations of C3 and C4 before and during pregnancy in systemic lupus erythematosus: association with disease flares and obstetric outcomes. J Rheumatol.

[32] Andreoli L, Bertsias GK, Agmon-Levin N, Brown S, Cervera R, Costedoat-Chalumeau N, Doria A, Fischer-Betz R, Forger F, Moraes-Fontes MF, Khamashta M, King J, Lojacono A, Marchiori F, Meroni PL, Mosca M, Motta M, Ostensen M, Pamfil C, Raio L, Schneider M, Svenungsson E, Tektonidou M, Yavuz S, Boumpas D, Tincani A (2017). EULAR recommendations for women’s health and the management of family planning, assisted reproduction, pregnancy and menopause in patients with systemic lupus erythematosus and/or antiphospholipid syndrome. Ann Rheum Dis.

